# Epithelial-mesenchymal transition: focus on metastatic cascade, alternative splicing, non-coding RNAs and modulating compounds

**DOI:** 10.1186/1476-4598-12-107

**Published:** 2013-09-23

**Authors:** Timur R Samatov, Alexander G Tonevitsky, Udo Schumacher

**Affiliations:** 1 SRC Bioclinicum, Ugreshskaya str 2/85, Moscow 115088, Russia; 2 The Institute of General Pathology and Pathophysiology, Russian Academy of Medical Sciences, Baltiiskaya str. 8, Moscow 125315, Russia; 3 P.A. Hertsen Moscow Research Oncology Institute, 2nd Botkinskii p. 3, Moscow 125284, Russia; 4 Department of Anatomy and Experimental Morphology, University Cancer Center, University Medical Center Hamburg-Eppendorf, Martinistr. 52, Hamburg D-20246, Germany

**Keywords:** Alternative splicing, Cell adhesion molecules, Epithelial-mesenchymal transition, Metastatic cascade, Non-coding RNAs, Small molecule compounds, Transcription factors

## Abstract

Epithelial-mesenchymal transition (EMT) is a key process in embryonic development and metastases formation during malignant progression. This review focuses on transcriptional regulation, non-coding RNAs, alternative splicing events and cell adhesion molecules regulation during EMT. Additionally, we summarize the knowledge with regard to the small potentially druggable molecules capable of modulating EMT for cancer therapy.

## Introduction

**Figure 1:**
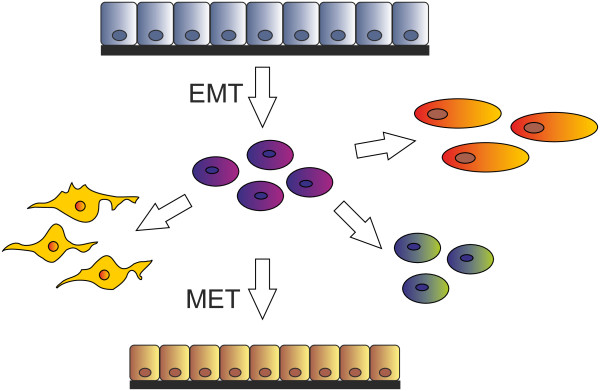
**Epithelial-mesenchymal transition.** Various mesenchymal cell types can be derived via EMT. The reverse mesenchymal-epithelial transition can generate secondary epithelia.

Epithelial-mesenchymal transition (EMT) is a multi-step morphogenetic process during which epithelial cells downregulate their epithelial properties and upregulate mesenchymal characteristics (Figure 1). Namely, static epithelial cells lose cell to cell junctions and as a consequence they lose apico-basal polarity to become migratory mesenchymal-like cells. This process of down-regulation of the epithelial phenotype mimics the normal developmental process of gastrulation, in which cells from the epithelial sheet of the ectoderm start to form the third germinal layer, the mesoderm, whose migratory cells are called mesenchymal cells. This process is therefore aptly called the epithelial-mesenchymal transition, which is currently classified into three subtypes [1].

Type 1 EMT is associated with the original embryonic development and also occurs during postnatal growth. The steps of this EMT type are specific and well-defined. Epithelial cells are cuboidal to cylindrical in shape and are in contact with each other via adherent and tight junctions. Primary migratory mesenchymal cells generated this way may potentially go through a reverse step to become epithelia again. This step is called the mesenchymal-epithelial transition (MET) and generates secondary epithelia in the developing embryo [2]. Differentiated cells in almost all organs in adults developed as a result of EMT-MET.

Type 2 EMT is initiated by injury and results in generation of fibroblasts to rebuild wounded tissues [3]. During inflammation fibroblasts and immune cells release cytokines and other pro-inflammatory factors as well as extracellular matrix proteins which results in stimulation of cells to undergo EMT. If inflammation pathologically persists, continuous EMT of normal epithelial cells can result in fibrosis and organ damage [4].

Oncogenic type 3 EMT enables epithelial cells to acquire invasive mesenchymal phenotype characteristics which are essential in metastatic spread [5]. Typical developmental EMT features are recapitulated in oncogenic EMT [6], however, they are less ordered and coordinated. As a result of this disordered EMT, hybrid phenotypes can often arise having the properties of both epithelial and mesenchymal cell types [7].

### Transcription factors regulating EMT

**Figure 2:**
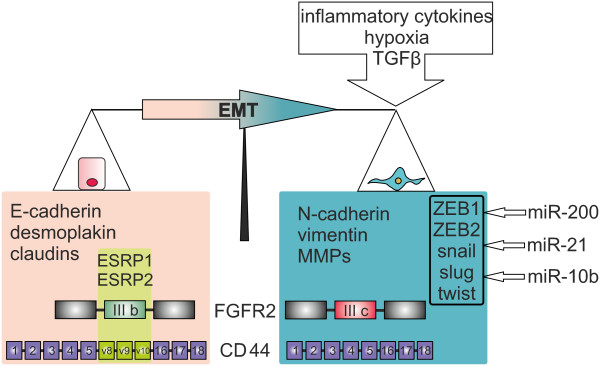
**Markers and regulators of EMT.** During EMT complex changes of mRNA expression level and alternative splicing of numerous genes occur. These changes are influenced by the tumor microenvironment, transcription and splicing factors and non-coding RNAs.

There are a number of transcription factors known to be involved in the regulation of EMT. The most characterized are ZEB 1 and ZEB 2, snail, slug and twist (Figure 2).

ZEB 1 and ZEB 2 are highly conserved zinc finger proteins which can directly bind to the promoter regions of target genes and thus repress the expression of E-cadherin and some other epithelial markers [8] and induce the expression of vimentin and a number of other mesenchymal markers [9]. ZEB 1 and 2 are induced by TGFβ, hypoxic conditions and inflammatory cytokines, factors which all initiate EMT. ZEBs play an important role in normal embryonic development and they are reported to be upregulated in many tumors [10].

Snail and slug belong to the snail family of transcription factors, with C-terminal zinc finger binding to E-boxes of the regulatory regions of target genes [11]. Snail factors repress E-cadherin expression by direct binding to its promoter and can also repress other epithelial proteins including desmoplakin and claudins. At the same time snail proteins activate expression of pro-invasive genes (vimentin, fibronectin, MMPs) promoting cell migration [12]. Like the two ZEB transcription factors, snail and slug can be induced by TGFβ, hypoxic conditions and other EMT-related signaling pathways [13]. Snail transcription factors are not present in normal epithelial cells, however they are found in the invasive front of tumors and considered to be prognostic factors for poor survival in a number of carcinomas [11].

The twist protein contains a basic/helix-loop-helix domain which provides for binding to DNA and dimerization. Its C-terminal end contains a “twist box” responsible for both transcriptional activation (e. g. for N-cadherin) and repression (E-cadherin) [14]. Regulation of genes by twist depends on its binding to other transcriptional factors, post-translational modifications, and choice of partner for dimerization. Twist is upregulated in human cancers and its abundancy increases during tumor progression. Its expression also correlates with higher tumor grade, invasiveness, and metastasis, cellular processes being considered as prognostic factors for enhanced tumor aggressiveness, tumor recurrence, and poorer survival [11].

Remarkably, there is a significant overlap in the regulatory signals of these transcription factors. Namely, expression of ZEB factors is regulated by snail [15,16]. Snail in turn also increases the stability of twist which then activates the transcription of slug [15,17]. This interaction network may play a role in spatial and temporal regulation of EMT.

### EMT and metastatic cascade

One of the classical models for cancer metastasis is Stephen Paget’s seed and soil hypothesis in which the tumor cell is the seed and the organ in which the metastasis grows is the soil [18]. This model implies that certain tumor cells have an affinity to the particular organ which provides a growth advantage to them. Thus the site of metastasis is dependent on the affinity of the tumor for the given microenvironment, which elegantly explains why some organs (lung, liver, bone marrow) are particularly prone to harbour metastases while others are not (intestine, skeletal muscle, skin). After passing the endothelial barrier, additional factors such as local growth factor production play a role in stimulating the growth of these evasive tumor cells [19]. According to this later expanded model, metastasis formation starts when the primary malignant cell divides and once the cell mass has reached the size of a few dozens cells, it sends out angiogenic signals, thus leading to the ingrowth of blood vessels into the newly formed tumor. As a next step, future metastatic cells have to free themselves from the primary tumor mass, have to degrade the surrounding extracellular matrix including the basement membrane, must enter the blood vessels and survive within the circulation (= the seed). Once they have reached the target organ of the future metastasis (= the soil), the tumor cell has to attach to the endothelium in this organ and has to migrate through it. When this process is accomplished, the metastatic cancer cell has - probably under the influence of local growth factors – to start to divide again in order to form a clinically detectable metastasis. Once proliferation has started, this metatsatic cycle resumes in order to spawn further metastases originating from a metastasis.

Different cell adhesion molecules (CAMs) play vital and opposing roles during this process. Due to their very epithelial nature cancer cells form more or less tight homologous epithelial cell to epithelial cell contacts at the site of the primary tumors. Molecularly this encompasses often homologous CAMs which are part of desmosomes, tight junctions and gap junctions (see Table 1). In addition, cell to basal lamina contacts (focal adhesions, hemidesmosomes) are formed from those cells directly adjacent to a basal lamina. In order to escape from the primary tumor, the proteins forming these junctions have to be down-regulated in order to allow cell migration. The contact of epithelial cells to the basal lamina not only hinders migration but also prevents cell death. If a normal epithelial cell looses the contact to the basal lamina, a special form of detachment-induced apoptosis, termed anoikis by Steven Frisch [20], is triggered as the cell’s integrins are detached from their ligands in the basal lamina. As mesenchymal cells do not necessarily have a direct contact to the basal lamina, they are not subject to anoikis and the EMT would therefore aid survival of the loosened cancer cells.

**Table 1:** Homologous CAMs

**Type of junction**	**Type of protein**	**Protein**	**Gene name**	**Function**
Desmosome	Cadherin (calcium-dependent)	Desmoglein 1, Desmoglein 2, Desmoglein 3, Desmoglein 4	*DSG1 DSG2, DSG3, DSG4*	Play important roles in cell adhesion, ensuring that cells within tissues are bound together. Cadherins behave as both receptors and ligands.
		Desmocollin 1, Desmocollin 2, Desmocollin 3, Desmocollin 4	*DSC1 DSC2 DSC3*	
	Catenin	Junction plakoglobin **(**JUP)	*JUP*	JUP can bind to the desmoglein I.
Tight junctions	Claudins	Claudin 1	*CLDN1*	The main component of the tight junctions
	Occludins	Occludin	*OCLN*	The main component of the tight junctions
	Cadherin	E-cadherin	*CDH1*	Loss of E-cadherin function or expression has been implicated in cancer progression and metastasis. E-cadherin downregulation decreases the strength of cellular adhesion within a tissue, resulting in an increase of cellular motility. This in turn may allow cancer cells to cross the basement membrane and invade surrounding tissues [21].
		F11 receptor (JCAM)	*JAM-1*	The ligand for the integrin LFA1, a platelet receptor
	Catenins	α-(E, N,T), β-, δ-catenins, γ-catenin (or Junction plakoglobin**,** JUP)	*CTNNA1 (CAP102), CTNNA2 (CAPR), CTNNA3 (VR22), CTNNB1, CTNND1, CTNND2, JUP*	Catenins belong to a family of proteins found in complexes with cadherin cell adhesion molecules. The primary mechanical role of catenins is connecting cadherins to actin filaments, specifically in these adhesion junctions of epithelial cells [22]. β-catenin may play a role in telling the cell to stop proliferating, as there is no room for more cells in the area.
				The role of catenin in EMT has also received a lot of recent attention for its contributions to cancer development. It has been shown that HIF-1α can induce the EMT pathway, as well as the Wnt/β-catenin signaling pathway, thus enhancing the invasive potential of LNCaP cells (human prostate cancer cells) [23]. As a result, it is possible that the EMT associated with upregulated HIF-1α is controlled by signals from this Wnt/β-catenin pathway [23]. Catenin and EMT interactions may also play a role in hepatocellular carcinoma. VEGF-B treatment of hepatoma carcinoma cells can cause α-catenin to move from its normal location on the membrane into the nucleus and E-cadherin expression to decrease, thus promoting EMT and tumor invasiveness [24].
				JUP protein is the only known constituent common to submembranous plaques of both desmosomes and intermediate junctions. JUP also associates with classical cadherins such as E-cadherin; in that context. Plakoglobin is O-glycosylated.
	Cingulin	Cingulin	*CGN*	Cingulin is specifically localized at tight junctions in epithelial cells, unlike ZO-1, which is also detected at adherens-type junctions in non-epithelial cells. Cingulin interacts with ZO-1 and several other tight junction proteins, in addition to interacting with actin and myosin [25,26].
	Actin	α-, β-, γ-actins	*ACTA1, ACTA2, ACTB, ACTG1, ACTG2*	Participates in many important cellular processes, including cell motility, cell division and cytokinesis, vesicle and organelle movement, cell signalling, and the establishment and maintenance of cell junctions and cell shape.
Gap junctions	Connexin (or hemichannel)	Connexins	*GJA1*, *GJC1, GJB4* etc.	Connexins are assembled in groups of six to form hemichannels, or connexons, and two hemichannels then combine to form a gap junction. The connexin gene family is diverse, with 21 identified members in the sequenced human genome.

The molecules forming homologous epithelial cell to epithelial cell tight contacts.

**Figure 3:**
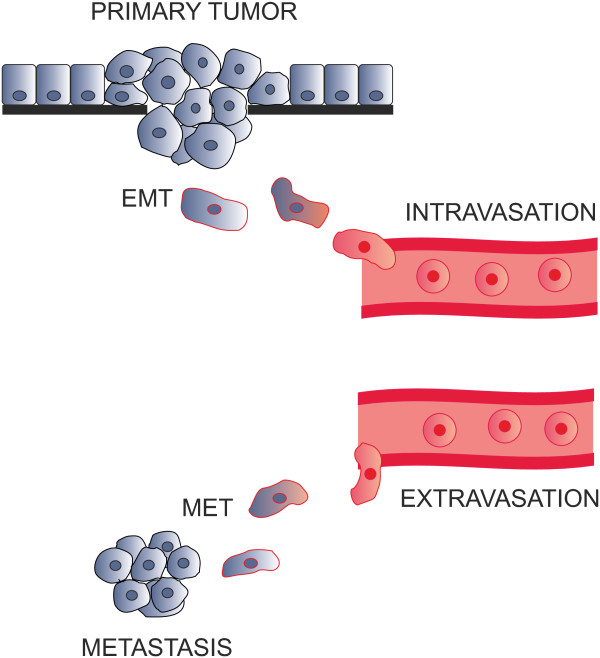
**The metastatic cascade.** In early stage of the metastatic cascade EMT enables migration and intravasation of tumor cells. After extravasation followed by MET metastasis is generated.

After EMT has enabled the tumor cells to migrate out of the primary tumor, they have to enter circulation and survive within it (Figure 3). Later, they must adhere to the microvascular endothelial cells at the site of the target organ and by this adhesion they have to communicate to the endothelial cells to open their cell junctions. This allows the passage of the cancer cell through the endothelium to the connective tissue space of the host organ. Again, CAMs mediate this process, however, these CAMs are different from those forming the intra-epithelial cell adhesion. Here, heterologous CAMs mediating cell adhesion between different cell types – tumor cells and endothelial cells - are important. Similarly to the mimicry of the EMT, cancer cells evading circulation mimic the leukocyte adhesion cascade (see Table 2). The CAMs and their ligands used in this adhesion are selectin glycoconjugate ligands, integrins and their extracellular matrix ligands, ALCAM and ICAMs. In contrast to the epithelial CAMs, which were down-regulated during EMT, these CAMs were up-regulated as part of the mesenchymal phenotype during EMT. These down- and up-regulations of cell adhesion molecule expression are governed by transcription factors which are important during gastrulation including twist, snail, slug, brachyury and ZEB 1 and ZEB 2.

**Table 2:** Heterologous CAMs

**Adhesion molecule (receptor)**	**Gene name**	**Localization and other information**	**Ligand**	**Gene name of the ligand**	**Localization of the ligand and other information**
Integrins					
Integrin alpha (CD11a)	*ITGAL (CD11A, p180)*	Integrin alpha combines with the beta 2 chain (ITGB2) to form the integrin lymphocyte function-associated antigen-1 (LFA-1). LFA-1 plays a central role in leukocyte intercellular adhesion through interactions with its ligands, ICAMs 1–3 (intercellular adhesion molecules 1 through 3), as a rolling and signaling molecule [27], and also functions in lymphocyte costimulatory signaling.	ICAM1 (CD54)	*ICAM1*	A member of the immunoglobulin superfamily. A glycoprotein which is typically expressed on endothelial cells and cells of the immune system.
Integrin beta-2 (CD18)	*ITGB2*				
					ICAM-1 can be induced by (IL-1) and (TNFα) and is expressed by the vascular endothelium, macrophages, and lymphocytes. ICAM-1 is a ligand for LFA-1 (integrin), a receptor found on leukocytes.
Integrin alpha M (ITGAM)	*ITGAM (CD11B, CR3A)*	Integrin alpha M is one protein subunit that forms the heterodimeric integrin alpha-M beta-2 (α_M_β_2_) molecule, also known as *macrophage-1 antigen* (Mac-1) or *complement receptor 3* (CR3). α_M_β_2_ is expressed on the surface of many leukocytes involved in the innate immune system. It mediates leukocyte adhesion and migration.			
Integrin alpha 4 (CD49d)	*ITGA4*	VLA4 (α_4_β_1_-integrin) is found on leukocytes and endothelial cells.	VCAM1 [28]	*VCAM1 (CD106)*	VLA4-interections support lymphocyte rolling in venules of the central nervous system in conjunction with P-selectin or can directly mediate rapid adhesion independent of P-selectin engagement [27].
Integrin beta-1 (CD29)	*ITGB1*		Fibronectin	*FN1*	Fibronectin is a high-molecular weight glycoprotein of the extracellular matrix [29]. Insoluble cellular fibronectin is a major component of the extracellular matrix. It is secreted by various cells. Fibronectin plays a major role in cell adhesion, growth, migration, and differentiation.
					Altered fibronectin expression, degradation, and organization are associated with a number of pathologies, including cancer and fibrosis [30].
Integrin		α_4_β_7_-integrin	MADCAM-1	*MADCAM1*	MADCAM-1 is a cell adhesion leukocyte receptor expressed by mucosal venules. It helps to direct lymphocyte traffic into mucosal tissues. It can bind both integrin alpha-4/beta-7 and L-selectin regulating both the passage and retention of leukocytes. Isoform 2 lacking the mucin-like domain may be specialized in supporting integrin alpha-4/beta-7-dependent adhesion strengthening, independent of L-selectin binding.
Selectins					
P-selectin	*SELP*	P-selectin is expressed on activated endothelial cells and platelets. Synthesis of P-selectin can be induced by thrombin, leukotriene B4, complement fragment C5a, histamine, TNFα or LPS.	PSGL-1 (P-selectin glycoprotein ligand-1)	*SELPLG (CD16)*	PSGL-1 is found on white blood cells and endothelial cells. PSGL-1 can bind to all three members of the selectin family however it binds to P-selectin with the highest affinity.
		P-selectin plays an active role in the rolling of leukocytes [27].			*see above* and: PSGL-1 was shown contribute to E-selectin-mediated initial leukocyte capture and rolling in vivo [31].
E-selectin (CD62E, ELAM-1)	*SELE (CD62E, ELAM-1)*	E-selectin is expressed on activated endothelial cells. E-selectin is not stored within the cell and has to be	PSGL-1	*SELPLG (CD16)*	
		transported to the cell surface. Synthesis of E-selectin follows shortly after P-selectin synthesis, induced by cytokines such as IL-1, TNFα and lipopolysaccharide (LPS). Shear forces can also affect E-selectin expression. E-selectin may interact indiscriminately with many glycoproteins and glycolipids [31].			
			ESL-1 (golgi glycoprotein 1)	*GLG1*	ESL-1 is a glycoprotein and a variant of a receptor for fibroblast growth factor.
					ESL-1 is a major E-selectin ligand on leukocytes [31].
			CD44	*CD44*	CD44 is expressed in a large number of mammalian cell types. This protein participates in a variety of cellular functions including lymphocyte activation, recirculation and homing, hematopoiesis, and tumor metastasis.
		E-selectin was shown to play a pivotal role in mediating cell–cell interactions between breast cancer cells and endothelial monolayers during metastasis [32].			
		E-selectin plays an active role in the rolling of leukocytes [27].			
					The contribution of CD44 is significant only at the later stages of the leukocyte recruitment cascade [31].
			GlyCAM-1	*GLYCAM1*	In breast cancer the splice variant 4 of CD44 was shown as a major E-selectin ligand in facilitating tumor cell migration across endothelial monolayers [32].
L-selectin (CD62L)	*SELL (CD62L, LAM1)*	L-selectin found on lymphocytes and preimplantation embryo. It plays important roles in lymphocyte-endothelial cell interactions.			GlyCAM-1 is a proteoglycan ligand expressed on cells of the high endothelial venules in lymph nodes.
			CD34	*CD34*	A cell surface glycoprotein which functions as a cell-cell adhesion factor. It may also mediate the attachment of stem cells to bone marrow extracellular matrix or directly to stromal cells.
					Cells expressing CD34 are normally found in the umbilical cord and bone marrow as hematopoietic cells, a subset of mesenchymal stem cells, endothelial progenitor cells, endothelial cells of blood vessels but not lymphatics (except pleural lymphatics). CD34 is also an important adhesion molecule and is required for T cells to enter lymph nodes. It is expressed on lymph node endothelia whereas the L-selectin to which it binds is on the T cell.
			MADCAM-1	*MADCAM1*	MADCAM-1 is a cell adhesion leukocyte receptor expressed by mucosal venules. It helps to direct lymphocyte traffic into mucosal tissues. It can bind both integrin alpha-4/beta-7 and L-selectin, regulating both the passage and retention of leukocytes.
			PSGL-1	*SELPLG (CD16)*	See above

The molecules which are responsible for the leukocyte adhesion cascade involved in the inflammatory response.

Circulating tumor cells (CTCs) are cells which have already separated from the tumor and entered the bloodstream. It has been demonstrated that the number of CTCs in blood is an important prognostic marker for breast [33], prostate [34], lung [35], bladder [36] and colon [37] cancer patients. CTCs are a heterogeneous population of tumor cells, some of them presumably underwent EMT and hence possess mesenchymal features, while others have not and still represent with a more epithelial phenotype. It has been demonstrated on groups of patients with distinct breast cancer stages that CTCs with mesenchymal markers are more typical for the late metastatic stage [38] and provide for the reliable prognosis of recurrence [39]. Another recently reported observation is that mesenchymal CTCs in patients with advanced cancer comprise multicellular clusters rather than single cells, in contrast to epithelial ones [40]. The authors explained this observation with the proliferation of the mesenchymal cell that has undergone EMT and after proliferation differentiated back into a more epithelially differentiated cell cluster which, however, seems contradictory to the typical individual mesenchymal phenotype. Alternatively, the authours hypothesized simultaneous EMT of a pre-existing cluster of CTCs in the bloodstream mediated by TGF-β released from platelets.

Despite the many efforts, the detection of CTCs still suffers from technical complexities and non-reliability of their isolation. These problems are due to the low abundance and heterogeneity of CTCs. The CellSearch and AdnaTest systems approved by the FDA in USA and by EU authorities, respectively, are based on the detection of epithelial markers. However, if the cells in the bloodstream are more of the mesenchymal phenotype, some important cell population might be missed by using these isolation techniques. Currently there is no reliable method and no defined list of markers for the detection of dedifferentiated EMT-derived CTCs [41].

### EMT and alternative splicing

More than 88% of human pre-mRNAs are alternatively spliced, thus generating protein diversity in an organism [42]. Alternative splicing events are regulated in a cell- and tissue type-specific manner, at different developmental stages or in response to extra-cellular stimuli and activation of specific signalling pathways [43,44]. As many of these processes occur during EMT, alternative splicing is of importance in EMT as well (Figure 2). Examples of the best characterized EMT-dependent alternatively spliced genes are FGFR2, CD44, p120-catenin and Mena.

The fibroblast growth factor receptor 2 (FGFR2) encodes for a fibroblast growth factor-activated transmembrane receptor tyrosine kinase and is the first discovered example of EMT-related alternative splicing [45]. The second half of the third extra-cellular immunoglobulin-like domain of the FGFR2 is encoded by one of two mutually exclusive exons IIIb (expressed in epithelial cells) or IIIc (characteristic for mesenchymal cells). The functional model suggests that epithelial cells expressing the FGFR2-IIIb form specifically interact with fibroblast growth factors produced by mesenchymal cells. Accordingly, the factors expressed by epithelial cells interact with FGFR2-IIIc [46]. These interactions have been demonstrated to be important during embryonic development and limb outgrowth and lung-branching morphogenesis. Remarkably, targeted down-regulation of mesenchymal-specific FGFR2-IIIc isoform was shown to decrease metastatic ability of TSU-PrI bladder cancer cells and to increase survival following in vivo inoculation in mice [47]. Interestingly, alternative splicing of a similar protein, namely FGFR3, is regulated by snoRNA HBII-180C [48]. This finding implies that non-coding RNAs regulate EMT through modulation of alternative splicing.

The CD44 gene encodes for a transmembrane protein which maintains tissue structure by mediating cell-cell adhesion [49]. The N-terminal domain of CD44 is extracellular and interacts with the extracellular matrix glycosaminoglycan hyaluronic acid (HA) facilitating the binding of a number of extracellular ligands. The formed complex initiates a downstream signaling cascade via the interaction of the intracellular domain with binding partners. The CD44 pre-mRNA comprises exons 1–5 at the 5’ end and exons 16–20 at the 3’ end that are spliced together into the standard isoform CD44s. This isoform is the smallest and is present on the membrane of most vertebrate cells. Between exons 5 and 16 are ten alternatively spliced variable exons (v1–v10). These alternatively spliced variants are longer than the standard isoform of CD44 and the proteins encoded by these variants show extended extracellular membrane-proximal regions which form a glycosylated stalk-like structure providing interaction sites for additional molecules [50]. The CD44E isoform containing exons v8–10 is predominantly expressed in epithelial cells correlating with the expression of E-cadherin [51]. Remarkably, induction of EMT in cultured cells resulted in a switch from CD44E to standard isoform, and expression of the latter was upregulated in human breast cancers and was correlated with the mesenchymal marker N-cadherin in these tumors [52].

Splicing is not a particular feature of CD44, indeed, CAMs in general are alternatively spliced. The most remarkable example is DSCAM (Down Syndrome Cell Adhesion Molecule) which has up to 18,000 splice isoforms [53]. This Ig-like receptor is involved in innate immunity and neural wiring and its gene is located on 21 chromosome.

p120-Catenin regulates cadherin stability and modulates Rho GTPase activity [54,55]. The isoforms containing exons 2 and 3 are expressed in mesenchymal cells. Epithelial cells skip these exons producing a shorter protein isoform. Consistently, EMT induces the expression of mesenchymal p120-catenin isoform [56]. Rho GTPases are known to regulate actin cytoskeleton and cell motility [57]. The full-length mesenchymal isoform of p120-catenin can bind RhoA GTPase, reducing its activity, and promote migration and invasiveness of the cells [58].

Mena (also known as Enah, mammalian enabled homolog of Drosophila protein Ena) is expressed in various cell types and regulates the branching actin filaments [59]. The isoform which contains the exon 11a is characteristic for epithelial cells and is not found in mesenchymal cells. Remarkably, it has been also found to be expressed in primary tumor cells but not in invasive tumor cells [60]. So far it is not clear what kind of functional implications this protein has on EMT.

Recently genome-wide approaches were used to determine EMT-related alternative splicing signatures [61]. It was shown that EMT-related extensive changes in alternative splicing are regulated by epithelial splicing regulatory proteins 1 and 2 (ESRP1 and ESRP2) [62]. These proteins are present in epithelial cells. Their siRNA-mediated knock down resulted in a splicing switch of FGFR2, CD44, p120 and Mena genes to mesenchymal phenotype. The reverse effect was observed when the ectopic expression of ESRP1 and ESRP2 was performed in mesenchymal cells.

Thus, there are clearly distinct profiles of alternative splicing which allow discrimination between epithelial and mesenchymal cell types.

### EMT and non-coding RNAs

MiRNAs are one family of small (20–22 nucleotides) non-coding RNAs. Their function is to regulate gene expression post-transcriptionally through binding to the sites which are perfectly complementary, or which may contain mismatches (“non-canonical sites”). These sites are located in 3’ UTRs, however recent reports demonstrate that miRNAs can also function through binding to other regions of target mRNAs [63,64]. By binding to target mRNAs, miRNAs play important roles in regulating diverse biological processes [65]. These processes include regulation of the EMT, in which various miRNAs are involved [66]. Remarkably, the regulatory miRNA-mRNA networks can be rapidly regulated [67]. It should also be mentioned that another recently reported interesting function of miRNAs, which may play a certain role in EMT regulation, is paracrine-mode intercellular signaling [68].

The miR200 and the miR205 families were shown to be highly associated with EMT and a strong correlation between the expression of the miR200 family and E-cadherin expression in different cell lines and epithelial tissues has been demonstrated [69,70]. During EMT, expression of miR-200 family is repressed by ZEB transcription factors. These factors in turn are the targets for miR-200 family thus comprising a double negative feedback loop [71]. It was shown recently that miR200c also regulates EMT through targeting fibronectin, moesin and other proteins that normally suppress cell migration and resistance to anoikis [72]. Moreover, the same lab found that miR200c targets a NF-κB-dependent neurotrophic tyrosine receptor kinase, which also suppresses resistance to anoikis, and this miRNA is down-regulated in highly aggressive triple negative breast cancers [73]. Other EMT-related downstream targets of the miR200 family are miR141 inhibiting TGFβ2 [74] and miR200a suppressing β-catenin (CTNNB1) [75].

The EMT-related transcription factors have been described as transcriptional regulators of miRNAs as well. For example, miR21 is abundant in various tumors and known to induce metastasis through EMT. The promoter regions of miR21 contain consensus E-box sequences comprising binding sites for ZEB1 [76]. Binding of ZEB1 induces transcription of miR21 [77]. MiR10b is also known to be associated with cell migration, invasion, and metastasis of breast cancer cells. It was shown that the transcription factor twist can bind to the E-box element close to the predicted promoter of miR10b and activate its transcription, thus promoting twist-mediated EMT [77]. Overall regulation of miR10b is complex and context dependent: ZEB1 increases the expression of miR10b in colorectal cancer cells but decreases expression in breast cancer cells [74]. Similarly, snail reduces the expression of miR10b in human breast cancer cells [77]. These data suggest that miRNAs can be considered as markers for EMT through the activity of EMT-related transcription factors.

MiRNAs were shown to be associated with the TGFβ signaling pathway. The TGFβ-mediated induction of EMT in mammary epithelial cells results in loss of tight junctions and cell polarity and up-regulates the expression of miR155 [78]. The target of miR155 is RhoA which is important for the control of actin cytoskeleton and cell invasion. RhoA contains three conserved regions which are potential binding sites for miR155 [78]. Down-regulation of RhoA leads to actin cytoskeleton rearrangements and increased cell motility [79].

The TGFβ-induced EMT in mammary epithelial cells also leads to the higher expression levels of miR29a and miR21 [78,80]. Ectopic expression of miR29a suppresses the expression of tristetraprolin and promotes to EMT in cooperation with the Ras pathway [80].

It has been demonstrated that miR9 regulates the mRNA encoding for E-cadherin [81]. Hence the increased expression of miR9 induced EMT in human mammary epithelial cells [81].

Remarkably, it has been demonstrated recently that circulating miRNAs in plasma of metastatic breast cancer patients can indicate their CTC status [82]. Circulating miRNAs are easier to isolate and handle than CTCs, which will probably make them prognostic markers of choice in future.

Long non-coding RNAs (lncRNAs) are an emerging class of RNAs longer than 200 nt. Our current understanding of their functional role is limited, however there are reports describing their involvement in the regulation of gene expression, chromatin remodeling, transcription, post-transcriptional RNA processing and cancer progression [83]. Metastasis-associated lncRNAs MALAT1 (8000 nt), HOTAIR (2200 nt) and ANRIL (3800 nt) are up-regulated in some tumors and can be potentially considered as EMT-related as they regulate EMT transcription [84]. More specifically, siRNA-mediated MALAT1 silencing resulted in down-regulation of the EMT-associated transcription factors ZEB1, ZEB2 and slug, and up-regulation of E-cadherin [85]. Moreover, MALAT-1 promoted EMT by activating the Wnt signaling pathway. It also has been demonstrated that MALAT-1 levels were significantly increased in primary tumors that subsequently metastasized comparing to those tumors that did not metastasize.

### Small molecule compounds modulating EMT

There are numerous kinases involved in TGF-β, Wnt, hedgehog and other signalling pathways regulating EMT and thus malignant progression. The basis of modern molecular targeted cancer therapeutics is the development of small molecule inhibitors capable of binding to the ATP-binding site of the dysregulated kinases. Thus the majority of the compounds affect EMT target kinases. For example, gefitinib and erlotinib, which are competitive inhibitors of EGFR, currently used for the treatment of advanced carcinomas, also demonstrate a protective effect against pulmonary fibrosis and hepatic fibrosis/cirrhosis, which supports their EMT-inhibiting activity [86,87]. Other well-known compounds are antiangiogenic drugs sorafenib and sunitinib that inhibit VEGFR and PDGFR, exhibit antifibrotic effects in the liver and have been demonstrated to inhibit EMT in in vitro cell culture models [88-90]. Compounds EW-7195 and EW-7203 target TGF-β type I receptor kinase/activin receptor like kinase-5 (ALK5) in a similar way, inhibiting TGF-β-induced EMT of mammary epithelial cells and preventing breast cancer metastasis to lung [91,92].

The drug BI 5700 directly inhibits kinase IKK2, a member of NF-κB signaling pathway whose activation causes EMT, and which has been demonstrated to revert EMT in metastasizing mouse colon carcinoma [93]. Another compound SL0101 targets ribosomal protein S6 kinase (RSK)-2 which is an important component of RON and TGF-β signaling pathways [94]. Both pathways regulate EMT, and inhibition of RSK-2 results in suppression of EMT-associated cell migration in an in vitro experimental system [94].

Interestingly, the complete reversal of EMT in vitro was achieved when a combination of the inhibitors of kinases TβRI (inhibitor SB431542) and ROCK (inhibitor Y27632) was used [28]. SB431542 down-regulate ZEB1 and ZEB2 levels, thus blocking mesenchymal gene expression of TGF-β-induced mesenchymal renal tubular epithelial cells. The Rho pathway inhibiting Y27632 was necessary to fully eliminate mesenchymal actin stress fibers.

Another type of small molecule modulator 4Ei-1 is a non-toxic nucleotide analogue which prevents the association of eIF4E and the mRNA cap. It inhibited cap-dependent translation in a dose-dependent manner in zebrafish embryos without causing developmental abnormalities and prevented eIF4E from triggering EMT in zebrafish explant model [95]. This compound can be considered as a potential anti-cancer drug and investigation of its effect on the tumors would be of a great interest.

Recently a high-throughput assay was developed to screen for small molecules interfering with EMT initiated by growth factor signalling using a model carcinoma reporter cell line NBT-II [96]. In this assay both cell growth and cell migration can be analysed simultaneously via time-course imaging in multi-well plates. The authors have validated several compounds targeting ALK5, MEK, and SRC kinases as efficient EMT inhibitors. This work highlights the growing interest in the small molecule compounds able to modulate EMT. Metastases are responsible for >90% of the cancer associated deaths. Therefore new strategies to prevent EMT which leads to metastases formation might be a promising novel approach in oncology.

## Conclusions

Epithelial-mesenchymal transition remains in the focus of a large number of researchers today due to its fundamental nature and important clinical implications. Non-coding RNAs and alternative splicing switches discussed in this review play important roles in EMT and cancer progression and can serve as markers for distinct epithelial or mesenchymal states of cells. Also, there are a growing number of discovered small molecules, belonging mostly to kinase inhibitors, which modulate EMT and have anti-cancer effect.

## Abbreviations

EMT: Epithelial-mesenchymal transition; MET: Mesenchymal-epithelial transition; TGFβ: Transforming growth factor β; CAM: Cell adhesion molecule; CTC: Circulating tumor cell; miRNA: MicroRNA; lncRNA: Long non-coding RNA.

## Competing interests

The authors declare that they have no competing interests.

## Authors’ contribution

TRS wrote the first draft of the article, AGT and US finalized the manuscript. All authors read and approved the final manuscript.
